# Chimeric antigen receptor T cell targeting B cell maturation antigen immunotherapy is promising for multiple myeloma

**DOI:** 10.1007/s00277-018-03592-9

**Published:** 2019-01-28

**Authors:** Tiantian Ma, Jing Shi, Huasheng Liu

**Affiliations:** Department of Hematology, The First Affiliated Hospital of Xi’an Jiaotong University, Xi’an, 710061 China

**Keywords:** Multiple myeloma, MM, Chimeric antigen receptor T cell, CAR-T, B cell maturation antigen, BCMA, Targeted immunotherapy

## Abstract

Multiple myeloma (MM) remains an incurable plasma cells malignancy because of its complex genetic heterogeneity and high relapse rate post immunotherapy. The encouraging results of chimeric antigen receptor T cell (CAR-T) targeting B cell maturation antigen (BCMA) immunotherapy clinical trials have shed light on curing MM in recent years. However, many therapeutic side effects limit the promotion and clinical use of this novel effective approach such as cytokine release syndrome, antigen escape, and neurotoxicity. We should make every effort to do further study about this immunotherapy to make it safer and effective. This review focusing on this topic clarifies the following contents: present status of MM treatment, effectiveness of CAR-T cells, features of BCMA, preclinical and clinical trials of BCMA CAR-T cells therapy, and existing problems and strategies. Hoping to provide a reference for the subsequent correlative clinical and research.

## Introduction

Multiple myeloma remains a highly incurable fatal hematopoietic malignancy and potentially curative and safer novel treatments are required. By systematically retrieving the research report and literature on this content and analyzing comprehensively, we find that the BCMA CAR-T cells immunotherapy shows great promise but still have many problems need to be resolved [1–3]. This review makes a comprehensive explanation about this therapy aiming to give some enlightenment to the clinicians and researchers.

## MM remains an incurable disease

Multiple myeloma is a malignant proliferative disease of plasma cells. Immunoglobulin-producing clonal plasma cells (PCs) proliferate and accumulate abnormally within the bone marrow (BM) can lead to hematopoietic insufficiency and lytic bone lesions. The excessive monoclonal immunoglobulins are deposited on the tissue, which can cause renal failure and/or amyloidosis and even cardiac dysfunction. Pathologic fractures, hypercalcemia, and opportunistic infections are also the common clinical manifestations of MM [4–8].

MM usually goes through the following stages: premalignant precursor condition, monoclonal gammopathy of undetermined significance (MGUS), smoldering MM (SMM), active MM, and end-stage plasma cell leukemia (PCL). This is the natural history of MM. In other words, MM is developed from an underlying precursor state, which is related to a series of cloning sequence evolution and a complex genetic background including deregulation of c-MAF, cyclin D1/D2, IRF4, and c-MYC, as well as mutations of TP53, CDKN2C, K-/N-RAS, and FAM46C [9, 10]. It is worth noting that all chromosomal aberrations, most transcriptomic changes and chromosomal mutations are already present in the stage of MGUS and SMM, which has been proved by a German fluorescence in situ hybridization (FISH) study [11].The BM accessory cells in the BM microenvironment also play an important role in the maintenance and progression of MM [8]. They secrete accessory growth factors/ligands such as IL-6, IGF-1, SDF-1α, B cell activation factor (BAFF), and a proliferation-inducing ligand (APRIL) and interact directly with MM cells, which mediate escape from immune surveillance leading to functional impairment of the host immune system as well as development of drug resistance. Moreover, Th1 cells, cytotoxic CD8^+^ T cells, macrophages, NK cells, Th2 cells, and dendritic cells (DCs) can also mediate protective immunity and promote tumor growth which is associated with the malignant transformation of the disease [12, 13].

The traditional treatment is to lower the malignant plasma cell load followed by maintenance treatment to prolong the patients’ life. And in the past decade, novel therapeutics such as new proteasome inhibitors, immune modulatory drugs, mAbs, and histone deacetylase inhibitors have been used in the clinic, which improve response rates and patients’ life quality obviously. Though some substantial improvement measures have been implemented in the therapy of multiple myeloma, this disease remains a largely incurable disease [1, 8, 14–16]. Nearly, the overwhelming majority of patients eventually relapse with increasingly refractory disease, which is really the main obstacle to the MM treatment and a large emotional burden for patients [15, 17–20]. And the huge genetic heterogeneity and the impact of bone marrow microenvironment on disease progression also make the disease hard to cure [21, 22]. So there is an urgent need to develop new treatment approaches for the MM patients. And achieving long-term responses, stable disease control and eventually cure is the therapeutic goals we pursue. Complex genetic heterogeneity poses great challenges to the treatment of MM and lead to poor outcome. However, the immunophenotype of MM cells is relatively homogeneous. A series of cell surface receptors and monoclonal immunoglobulins are expressed stably and uniformly on nearly all MM cells, which provide immunotherapeutics with potential targets and make the approach promising [23].

Therefore, it is feasible to develop a novel next-generation effective immunotherapies targeting the specific cell surface molecules to inhibit MM cells growth and eliminate the promoting factors in the BM microenvironment, which may allow the potential cure of MM.

## CAR-T cells targeting BCMA immunotherapy shows promise

In recent years, there has been much focus on the MM immunotherapies such as immunomodulatory drugs (IMiDS), allogeneic stem cell transplants (allo-SCT), monoclonal antibodies, immune checkpoint inhibitors, chimeric antigen receptor (CAR) T cell therapy, dendritic cell (DC)-based vaccines, cytokine-induced killer cells (CIKs), and tumor infiltration lymphocytes (TILs) [7, 10, 24, 25]. However, the anti-BCMA CAR-T cells seem to be the most promising one so far.

### CAR-T cells therapy is a novel effective approach

**Fig. 1:**
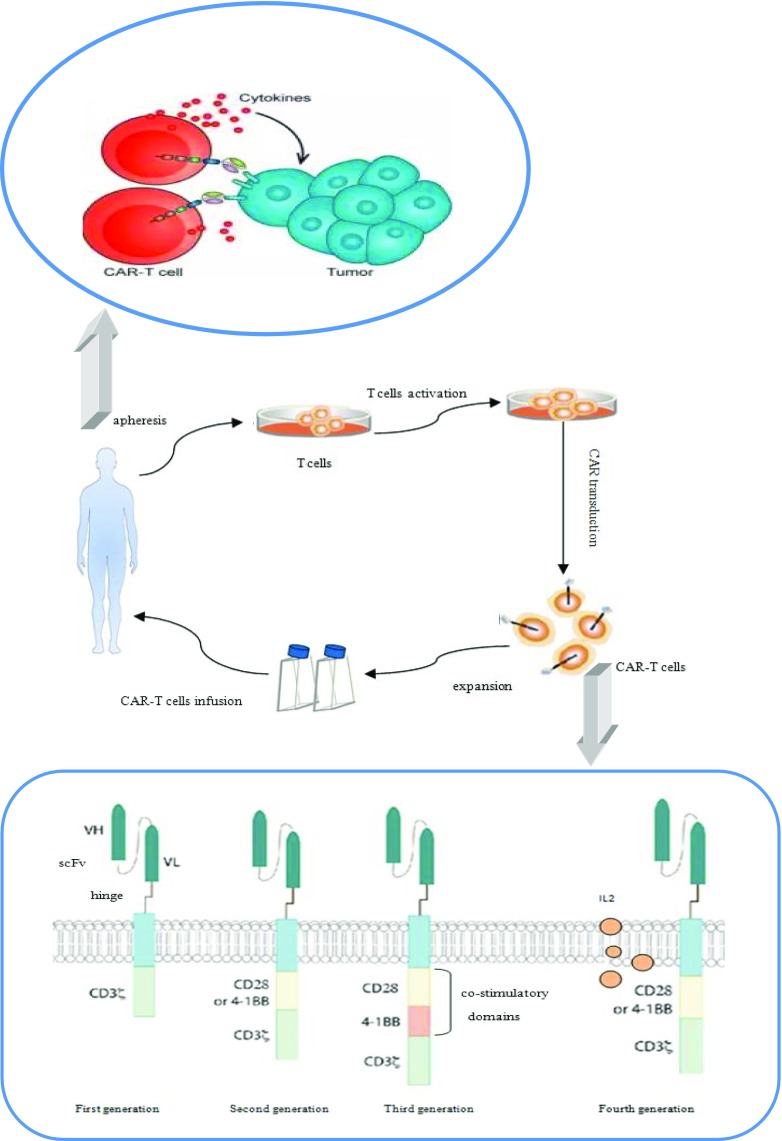
Mechanism and structure of CAR-T. The patients’ own T cells are collected to be modified with specific CAR which is transduced by lentiviral vectors or retroviruses. Then the CAR-T cells are infused back to the patients to identify, bind and lyse the targeted cells. The recombined T cells consist of an extracellular targeting region and various intracellular signaling domains which are connected by a hinge transmembrane region. The extracellular section is a single-chain variable fragment (scFv) and the intracellular section is the activation domains which include CD3ζ chain and one (second or fourth generation CAR) or two (third generation CAR) co-stimulatory domains such as CD28, 4-1BB, and CD27 which can enhance the signal transduction and affect their functions. The fourth generation CAR can also express cytokine such as IL-12 to promote the proliferation of T cells

Chimeric antigen receptor-expressing T cells immunotherapy is a novel promising therapy which combines the target specificity of monoclonal antibodies and the cytotoxicity of T cells. In this approach, the patients’ own T cells are collected to be modified with specific CAR which are transduced by lentiviral vectors or retroviruses that is CAR-T cells. Then the CAR-T cells are infused back to the patients. The CAR modified T cells do not rely on endogenous activation and co-stimulation but receive supra-physiologic stimulatory signals through the CAR. The recombined T cells can identify, bind, and lyse the targeted cells, as well as proliferate, which shows a specific and durable effect. Both CD4^+^ and CD8^+^ T cells show capacity of specific target activation and the percentage of CD4^+^ T cells is higher than CD8^+^ T cells. The CAR-T cells consist of an extracellular targeting region and various intracellular signaling domains which are connected by a hinge transmembrane region commonly derived from CD8 or IgG4. The extracellular section is a single-chain variable fragment (scFv) derived from a monoclonal antibody which can identify and bind the specific tumor-associated surface antigen (TAAs) in a non-MHC-restricted manner. The intracellular section is the activation domains of CAR-T cells which include CD3ζ chain and one (second or fourth generation CAR) or two (third generation CAR) co-stimulatory domains such as CD28, 4-1BB, and CD27 which can enhance the signal transduction of CAR-T cells and affect their functions. The fourth generation CAR can also express cytokine such as IL-12 to promote the proliferation of T cells [5, 26–30] **(**Fig.1). It has been proved that CD28 CARs have stronger activity of producing cytokines compared with 4-1BB CARs but lack of persistence. So far, there is no evidence showing that the third generation CAR-T cells have better efficacy than the second generation CAR-T cells [31, 32]. CAR-T cells can identify the target antigen even expressed at low level on the cell surface though the exact number of molecules that can induce clinical response has not been sure. On the one hand, this feature can improve the sensitivity of the treatment. On the other hand, it also shows the potential off tumor/on target toxicity which is usually associated with the choice of the target antigen. The introduction of CAR-T cells therapy has revolutionized immunotherapy and tumor treatment as a whole [33]. CAR-T cell targets include BCMA, CD19, KLC, CD138, CS-1, CD38, NKG2D ligands, and CD44v6 [28, 34]. The recent success of CAR-T targeting CD19 in acute lymphoblastic leukemia has made people be more confident with this new technology and great interest has been spurred in broadening the technology to other relapse/refractory hematological malignancies. MM is one of the hot fields of study [35–37]. It has been proved that anti-CD19 CARs can eradicate normal B cells [38]. But because of the rare expression of CD19 on the malignant plasma cells of MM, the CD19 CAR-T has limited clinical use in MM therapy. So there is an urgent need to find a new suitable target for MM.

### BCMA is an excellent target for MM CAR-T therapy

Recently, attention has been shifted to the B cell maturation antigen (BCMA) as an ideal CAR target for MM immunotherapy.

BCMA or CD269, the tumor necrosis factor receptor superfamily member 17 (TNFRSF17), plays a central role in regulating B cell maturation, differentiating into PCs and mediating the survival of PCs [27, 39–42]. It is a kind of type III transmembrane protein lacking a signal-peptide but containing cysteine-rich extracellular domains. It is expressed exclusively on B cell linage cells especially on the interfollicular region of the germinal center, plasmablasts, and differentiated PCs, but is not present on naïve B cells or hematopoietic stem cells. BCMA can also be detectable on plasmacytoid dendritic cells and some memory B cells though the expression intensity is much lower than CD138 ^+^ PC (more than a tenfold difference) [43]. And there is no known expression on normal essential solid tissue. It really has a very restricted expression pattern [44–46]. In the patients of MM, BCMA is uniformly expressed on malignant plasma cells at high levels and high specificity, which has been convinced by gene and protein expression spectrum. The intensity of BCMA expression is associated with the loss of BAFF-R, that is, the downregulation of BAFF-R always follows the upregulation of BCMA [47]. And the expression level of BCMA increases with the MM progression, from normal to MGUS to SMM to active MM. These expression features make BCMA an excellent target for MM immunotherapy [44, 46]. On the other hand, the donor derived anti-BCMA mAbs can be detectable in the patients receiving allogeneic transplant and the graft-versus-MM response can be seen after the infusion of donor lymphocytes, which further prove that BCMA is really a promising target for MM therapy [48]. It has been proved that the BCMA plays an important role in MM pathogenesis and pathophysiology. In the chromatin immunoprecipitation, BCMA is co-immunoprecipitated with interferon regulatory factor 4(IRF-4) which is a main transcription factor mediating the survival of MM cells. That shows the potential function of BCMA in oncogenesis [49]. BCMA can promote the survival of B cells at different stages of development by engaging APRIL and/or BAFF to activate the growth and survival signaling cascades [12]. As for MM patients, the signaling cascades can also provide a substantial anti-apoptotic signal for the malignant MM plasma cells, which is an important factor of the disease progression [50].

In summary, BCMA is really a perfect choice for the target of the CAR-T cells in MM.

### Preclinical and clinical trials of anti-BCMA CAR-T cells have encouraging results

The efficacy of BCMA-CAR T cells has been proved by preclinical studies in murine xenograft models in which MM cells in the mouse can be completely eradicated by anti-BCMA CAR-T cells and the survival of the mouse model is prolonged significantly. The vitro study also suggests the potential ability of BCMA-CAR to overcome the drug resistance induced by BM microenvironment in relapse/refractory MM.

Up to now, there have been a number of clinical trials conducted utilizing the anti-BCMA CAR-T cell therapy in multiple myeloma (MM), some of which are summarized in Table 1.

**Table 1:**
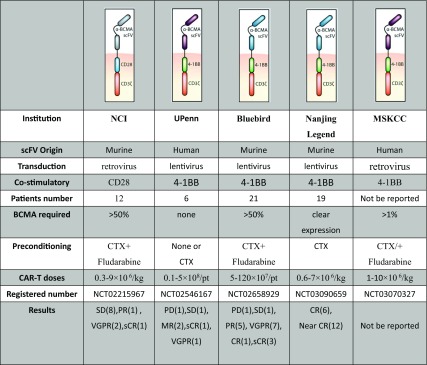
Compare of clinical trials about BCMA CAR-T cells

*SD*, stable disease; *PR*, partial response; *VGPR*, very good partial response; *sCR*, stringent complete response; *PD*, progressive disease; *MR*, minimal response; *CR*, complete response

The first-in-human clinical trial investigating the efficacy and safety of the 2nd–generation anti-BCMA-CAR with CD3/CD28 signaling domains was conducted by Kochenderfer et al. in 2016. And this group had reported their work on a novel CAR targeting BCMA in multiple myeloma and demonstrated the efficacy against myeloma cells in preclinical models about 3 years ago [44]. In this clinical trial, the BCMA scFv was derived from a murine hybridoma and the cells were transduced with the γ-retroviral vector. Only MM patients with uniform BCMA expression by either IHC or flow cytometry and normal major organ function were treated. It was designed as a phase I dose-escalation trial consisted of 12 relapse/refractory MM patients who had received a short course of conditioning with three doses of 300 mg/m^2^ cyclophosphamide (CTX) and three doses of 30 mg/m^2^ fludarabine which were administered daily on days − 5, − 4, and − 3 before CAR-BCMA T cell infusion on day 0. The 12 enrolled patients were treated with a single dose of CAR-BCMA T cells respectively and the dose escalation was at four different levels which were 0.3, 1, 3, and 9 × 10^6^ CAR-T cells/kg body weight. In the two lower dose level groups, limited antimyeloma activity and mild toxicity occurred. A patient had a transient partial response (PR) of 2 weeks duration and other five patients had responses of stable disease (SD). Better clinical responses were seen at the two higher dose levels. In the third level group, a patient had a very good partial response (VGPR) of 8 weeks duration and three other patients obtained responses of stable disease. And at the maximum dose level, there was an ongoing VGPR at 26 weeks and a stringent complete response (sCR) lasting 17 weeks. All the patients experienced cytopenia and other different kinds or grades of toxicities. The higher level group had more serious toxicity such as cytokine release syndrome (CRS) with symptoms including fever, hypotension, dyspnea, and prolonged cytopenia [50]. This clinical trial provides the first proof of the novel approach and clearly reveals the antimyeloma activity power of the anti-BCMA CAR-T cells. It also makes a benchmark for subsequent trials, which is really a critical step toward chemo-free treatments for MM.

Another ongoing phase I dose-escalation study about the second generation 4-1BB-CD3ζ anti-BCMA CAR-T cells was conducted by Cohen et al. from the University of Pennsylvania. The BCMA scFv origin was fully human and the cells were transduced with the lentiviral. The relapsed/refractory MM patients included in this trial needed adequate renal, hepatic, cardiac, and pulmonary function but no specific BCMA expression level. They were designed to receive split-dose infusions of BCMA CAR-T cells (10% on day 0, 30% on day 1, and 60% on day 2). Three cohorts were planned: (1) 1–5 × 10^8^ CAR-T cells alone, (2) CTX 1.5 g/m^2^ + 1–5 × 10^7^ CAR-T cells, and (3) CTX 1.5 g/m^2^ + 1–5 × 10^8^ CAR-T cells. And until the results were presented in 2016, six patients had been treated in cohort 1. Among the six patients, the one who received the lowest dose of 1.8 × 10^8^ CAR-T cells had progressive disease (PD). The three who received maximum dose, 5 × 10^8^ CAR-T cells, had minimal response (MR) of 2 months, SD lasting 2 months, and ongoing MR 1 month post-infusion respectively. One of the patients who received 2 × 10^8^ CAR-T cells had VGPR progressed at 5 months because of BCMA expression loss and the other achieved ongoing sCR for more than 7 months. In this trial, side effects such as CRS and posterior reversible encephalopathy syndrome (PRES) were observed. The preliminary test results demonstrated the effective clinical activity of this approach even without lymphodepleting conditioning and the promising CAR-T cells expansion in vivo. The trials of other cohorts are ongoing [51].

Bluebird Bio and partner Celgene recently have reported their multi-center phase 1 dose escalation trial results of the BCMA CAR-T product bb2121 which consists of autologous T cells transduced with a lentiviral vector encoding a novel CAR engineered with a murine-derived anti-BCMA scFv and a 4-1BB costimulatory motif. bb2121 can recognize tumor cells expressing as little as 222 BCMA molecules per cell and it shows rapid and sustained elimination of the tumors and 100% survival in the pre-clinical study [45]. This trial enrolled relapsed and/or refractory multiple myeloma (RRMM) patients with ≥ 3 prior regimens and ≥ 50% BCMA expression. Before the infusion of bb2121, patients need to undergo lymphodepletion with fludarabine (30 mg/m^2^) and cyclophosphamide (300 mg/m^2^) daily for 3 days. The planned dose levels were 5.0 × 10^7^, 15.0 × 10^7^, 45.0 × 10^7^, 80.0 × 10^7^, and 120 × 10^7^ CAR-T cells. As of May 4, 2017, 21 patients had been infused with bb2121, and 18 patients were evaluable for initial clinical response. No dose-limiting toxicities (DLTs) and no treatment-emergent grade 3 or higher neurotoxicities had been observed. Fifteen of 21 patients had cytokine release syndrome (CRS), but primarily grade 1 or 2. Grade 3 CRS was only been seen in two patients of the two higher dose groups. Overall, bb2121 had relatively low incidence and grade of CRS and neurotoxicity compared with other BCMA CAR-T products. One of three patients in the first level group had PR and in the second group with six patients, there were two sCR, one VGPR and one PR. Better results were seen in the nine patients of the third level group with one sCR, five VGPR, and two PR. And three patients in the fourth group had CR, VGPR, and PR respectively. There was one death in this trial due to cardiopulmonary arrest which was assessed as unrelated to bb2121 infusion. This trial is ongoing at higher dose levels. The initial data have indicated the safety and efficacy of the BCMA CAR-T cells bb2121 [52].

Nanjing Legend is also conducting a clinical trial of 4-1BB-CD3ζ CAR-T cells targeting BCMA, LCAR-B38M, which really have encouraging results. The BCMA scFv was derived from a murine hybridoma and the cells were transduced with the lentiviral. Nineteen RRMM patients were included in the trial and the median number of infused cells was 4.7 (0.6~7.0) × 10^6^/kg. Six out of the seven patients who were followed up for more than 6 months achieved CR and minimal residual disease (MRD)-negative status. The other 12 patients who were followed up for less than 6 months were observed with a progressive decrease of M-protein and expected to eventually meet CR. CRS was seen in 14 patients including nine cases of grade 1, two cases of grade 2, one case of grade 3, and one case of grade 4 [53].

Memorial Sloan Kettering Cancer Center (MSKCC) have recently opened a phase I trial using MKSCC hu BCMA-CAR which is transduced with retrovirus and includes a fully human BCMA scFv and a 4-1BB co-stimulatory domain. In this trial, there is no strict restriction of the BCMA expressing levels and the BCMA positive (> 1%) is eligible. The pre-condition is cyclophosphamide for the first cohort, with patients treated in subsequent cohorts additionally receiving fludarabine. This trial is ongoing and the results have not been reported yet.

Overall, the reported results of these clinical trials currently show great promise to the cure of MM.

## Problems and strategies

Though BCMA CAR-T cells have shown great success in the therapy of MM, there are still many problems we need to solve.

Cytokine release syndrome (CRS) is a main critical toxicity in various CAR-T cell clinical trials which is associated with the expansion and activation of CAR-T cells. The dramatic elevation in pro-inflammatory cytokines such as IL-6, IL-10, IFN-γ, and GM-CSF is the main hallmark of CRS. Most people have mild flu-like symptoms such as fever and mild hypotension. In more severe cases, hypoxia, hypotension, capillary leak, and coagulation disorders can be observed. And these may lead to fatal multi-organ dysfunction and even evolve into fulminant hemophagocytic lymphohistiocytosis (HLH). Neurotoxicity, termed CAR-T cell-related encephalopathy syndrome (CRES), which can occur concurrently with or after CRS, is the second most common adverse event. Intensive monitoring and prompt management of toxicities are really needed [54–57]. Antigen escape and the persistency of BCMA-CAR T cells are also problems should be paid attention to. It has been observed that MM relapse with reemergence of BCMA^+^ or BCMA^−^ malignant cells after BCMA-CAR therapy and the BCMA^+^ relapse is the majority [50]. Another potential shortcoming of BCMA CARs is that they can target the soluble BCMA in the serum. Whether the interaction between the BCMA CAR-T cells and BCMA^+^ MM cells can be affected is not very clear yet. But it has been proved that the BCMA protein in the culture media cannot block their mutual recognition and the anti-BCMA CAR-T cells can also inhibit the growth of MM cells in the mouse model which has had soluble human BCMA in vivo [4].

To date, there have been some good ways and ideas to control the therapeutic side effect and enhance the CAR-T cells effect with the continuous progressions of study. The following ideas can provide a good reference.

Traditional management of CRS is glucocorticoid used in the whole body. However the effect of the CAR-T cells can be inhibited after long-term use of glucocorticoid. To solve this problem, the anti-IL-6 receptor (IL-6R) antibodies are a new approach such as tocilizumab. However, the relationship between the IL-6R and the CAR-T cell anti-tumor effect has not been clear so far [54, 58]. Anti-IL-6 therapy is also recommended for patients with grade ≥ 1 CRES with concurrent CRS and for grade ≥ 2 CRES not associated with CRS, corticosteroids are preferred. What’s more, the CAR-T cell therapy associated TOXicity (CARTOX). Working group has been formed to monitor, grade, and manage the acute toxicities occurring in patients treated with CAR-T cells [56].

As for the antigen escape, dual-targeting CAR-T design which allows the CAR-T cells to recognize two antigens at the same time is a good way to increase targetable tumor antigens and reduce the risk of antigen-negative disease escape. For example, AUTO2, a CAR-T product targeting both BCMA and TACI, shows a potential to overcome this challenge [59]. Also, the compound CAR targeting both BCMA and CS1 which has roles in myeloma parthenogenesis and specific immune cell activities, that is BC1cCAR, is able to augment the anti-tumor response compared to a single BCMA or CS1-CAR and can eliminate any BCMA^−^CS1^+^ potential relapse [60, 61].

The potency of BCMA CAR-T cells can be augmented with optimizing the CAR-T cells design. For example, we can change the costimulatory domains to enhance reactivity and the humanized binding domains can reduce the immunogenicity to some extent. Adjusting the proportions of CD8^+^ and CD4^+^ T cells moderately may also have impact. Suicide genes such as herpes simplex virus thymidine kinase (HSV-tk) and inducible caspase 9 (iCas9) can be added into the structure of CARs to eliminate the CAR-T cells when severe side effects of the treatment appear. The inhibitory CARs (iCARs) which has a surface antigen recognition domain linked to the T cell inhibitory receptors of either PD-1 or CTLA-4 is also a strategy to avoid unwanted T cell reactivity. The iCARs response can be inhibited by 90% when it recognizes targets expressing both of the antigens [62]. And making the CAR-T cells express certain chemokine receptors can induce the cells to home to the MM microenvironment directly which may induce the on target/off tumor toxicity [62–65]. It is worth noting that the NCI and Blubird bio trials use a murine hybridoma-derived scFv, while the UPenn and the newly opened MSKCC studies utilized a human library screening approach to identify scFv’s. It is observed that expansion of CAR-T cells is limited by the development of host anti-murine scFv immune responses. And the host anti-CAR immune responses against human derived CARs may be less than murine hybridoma-derived which may also mitigate the necessity of preconditioning [8, 50, 51]. Furthermore, a bicistronic construct including a human single-chain variable fragment-derived BCMA-CAR and a truncated-epithelial growth factor receptor (EGFR) marker are under clinical investigation recently which shows rapid expansion (>10,000-fold, day 6), eradication of large tumor burden, and durable protection [66]. Other surface markers such as CD20 and c-myc have also been introduced in the CAR-T cell that can later allow the elimination of the CAR-T cell using mABs [62].

GSK2857916 is a novel antibody–drug conjugate (ADC) targeting BCMA with afucosylated Fc linked to monomethyl aurastatin F via noncleavable linker. These two approaches have different antigen binding sites which show that the sequential treatments may provide even more robust anti-MM effect. And then the persistence of BCMA-CAR T cells can be improved [4, 43]. Transmembrane activator and calcium modulator and cyclophilin ligand interactor (TACI) are also expressed on MM cells and it can also bind APRIL and BAFF, which is the same as BCMA. So we can enhance the anti-BCMA CAR-T cells effect by blocking the pathway of B cells activation through TACI binding APRIL and BAFF. And there has been some effective products. LY2127355 is a monoclonal antibody which can neutralize BAFF. And the combination of LY2127355 and bortezomib has shown good safe profile in MM patients. The first therapeutic APRIL neutralizing monoclonal antibody can inhibit the growth of MM cells in vitro and in vivo, which has been testified by preclinical study. And Atacicept, a kind of fusion protein neutralizing BAFF and APRIL, can also induce the apoptosis of MM cells and suppress the proliferation [67, 68].

Homology-directed recombination (HDR) is a kind of gene editing technique that can induce the functional gene cassettes to target loci. The simultaneous gene delivery/knockout can be achieved in this way. We can use HDR gene editing to deliver a CAR combined with other modifications of the T cell genome which can enhance the potential of CAR-T cells therapy and expand the treatment scope. For example, CCR5 can code the co-receptor of HIV-1 and its’ null mutations can make the T cells receive the resistance to the infection of the most prevalent strains of HIV-1. So using HDR to deliver BCMA-CAR into the CCR5 locus to produce a kind of CCR5 negative anti-BCMA CAR-T cells is probably the best way for the HIV^+^ MM patients. TCR-deficient CAR-T cells can be produced by introducing a CAR expression cassette into the T cell receptor alpha constant (TRAC) locu, which may allow an allogeneic CAR-T cells to be used in the patients who cannot generate autologous T cells [69, 70].

## Conclusions and future perspectives

BCMA CAR-T cells immunotherapy is really an effective approach for relapse/refractory MM which has been testified not only in theory but also in various clinical trials. Though there are still many problems and challenges need to be resolved, this novel therapy shows great promise for the cure of MM indeed. Future directions include optimizing the design of anti-BCMA CAR-T cells and finding the most appropriate infusion way and dose level. And we also need to study the relationship between BCMA expression levels and the response rates and durability of CAR-T cell therapy. Furthermore, it will be meaningful to clarify the appropriate applicable types and the physical conditions of MM patients. Believing MM can be cured 1 day with reasonable and efficient use of the anti-BCMA CAR-T cells therapy.
